# Foil and Fillings

**Published:** 1872-07

**Authors:** E. L. Hunter


### ARTICLE II.

### *Foil and Filling.*

BY E. L. HUNTER, D.D.S.

Alexander Von Humboldt said, "How much we want facts." Is it not time for our peculiar profession to cry out with this great thinker? To-day we abandon a principle, to-morrow we accept it. We lay aside practical conveniences, with which use has made us skilful to pioneer amid new theories. May we not ask if it is not often *because they are new*. It is all well and pleasant to see the populous city of the West, which was fathered by a pioneer, seemingly but yesterday; but when we have to hew out improvements through the perishing flesh and blood of our fellow creatures we should feel the light of facts before we step on so ruthlessly with the so-called "roped strides," in order that we may not have to remove our work with stricken consciences.

When thought, with years of labor, has made one skilful with soft foil, should he not have a care how he abandons it for something with which he is entirely unskilled? Surely a well made soft foil filling is far better than many hundred of the adhesive ones we have seen; for instance some made on the approximal surface of incisor teeth where the space was not sufficient to pass an uncrumpled pellet of adhesive foil to the cavity. Under such circumstances soft foil, fresh and carefully prepared and manipulated would have been abundantly effectual in preventing further decay during life. When either the palatine or labial wall of these cavities is unduly thin, we cut it away and use adhesive foil; when, however, such is not the case we use soft foil folded with cards—not touching it with the hands—to a width equal that of the cavity, and carry it against the cervical wall with great force, condensing each fold as it is carried to its place as perfectly as we would each pellet, were we using adhesive foil. Where the cavity will no more than admit the point of the plugger to pass freely to the bottom, we fill the remainder with a number of small pellets of adhesive gold; when the whole is properly condensed and finished it is inferior for that locality to no adhesive filling which can be made, but superior to one which could be made there, because the adhesive pellets would be partially condensed in their introduction, and therefore not so servicable, to say nothing of the difficulties attending the proper forming and filling a retaining point, without which we are almost hopeless of making at any time a strictly first-class adhesive filling. We use these retaining points merely for the purpose of tacking our first pellets in position while we mould our gold to a cavity, upon the general formation of which, and not retaining points, we place our hopes for stability.

The finishing of a soft foil filling with adhesive pellets may be surely permanent, when a hole with parallel walls is left to be filled. It is quite an advantage to finish in this way, since if each vertical fold of soft foil is properly condensed it will be impossible to enter a wedge in the centre, and the last folds introduced would be in a hole so small that they would have necessarily to be at right angles to the former ones. This with soft or non-adhesive foil would be liable to come away at some time, at last a portion of the cavity opened to decay. Whereas in the orthodox manner of using this foil, all the folds would be parallel and the finishing done through a hole made in the centre, with the same foil, in which case the same crumbling is always liable, to a greater or less extent, in the centre of the filling, leaving a roughened surface. But the grafting of adhesive upon non-adhesive gold in the approximals of bicuspids and molars where the grinding surface has been cut away, seems more a convenience than a utility, except it be where anchorage is taken in the fissure of the grinding surface and under the cusps; for suppose those who use soft foil in the commencement of these fillings, do so to get their work above water, avoid retaining points, or furnish something to stick their pellets to. A direct approach to every part of the cervical wall has been opened through the grinding surface, thus enabling retaining points to be made and filled, and adhesive pellets condensed with ease on any part of it; than which nothing could be more effectual. Why use the soft foil in any part of it, unless it be that extreme sensitiveness prevents the formation of retaining points?

As many persons handle their gold in preparing it, thus rendering it less serviceable and harder to work, I give my simple method with the assurance that it works very nicely. I find it of much service to know what width to cut my strips of foil, as the numbers vary, since if too wide the pellets will be too bulky to be properly condensed. If too narrow more time than necessary will be occupied in making the filling. Under ordinary circumstances we use Nos. 4, 6, 10, or 20, and cut the strips from a quarter of an inch to a half sheet, as the No. varies from 20 to 4. It being fresh, adhesive, or properly annealed, we lay the strips of foil on a quire of bib. paper, or clean chamois skin, then place lengthwise on the strips near the edge a medium sized, bright knitting needle, press it down, then place under the edge of the strips a very small knitting needle, rotate it above the other needle, then the large above the small, and so on until the whole is rolled on the two needles, when the needles one at a time may be withdrawn leaving an accurate cylindrical wall with no rucks; cut this into sections from one-eighth to three-fourths of an inch in length, and we have the gold in such form as will enable us to condense as uniformly as though we were working the heavier Nos. without folds. A size of needles should be used which will make a roll but little greater in diameter than the cavity to be filled, so that it may be neatly folded down in the cavity. If each fold is struck over its whole surface with a small point, either serrated or smooth, either with hand or mallet force, when the cavity is full and the filling polished, no anxiety will be felt for Nos. 240, or 480. We are constantly feeling that our want is more a skilful manipulation of the materials and instruments at hand, than new materials and new instruments. Very many of the mouths we look into proclaim aloud a want either of skill or honesty.

---

